# Data-driven Huntington’s disease progression modelling and estimation of societal cost in the UK

**DOI:** 10.1098/rsos.240824

**Published:** 2024-11-20

**Authors:** Andrew Pollard, Danica Greetham, James Myatt, Hugh Rickards, Cath Stanley, Dave Dungate

**Affiliations:** ^1^ Hybrid Intelligence, Capgemini Engineering, Stevenage, UK; ^2^ Huntington’s Disease Association (England and Wales), Liverpool, UK; ^3^ University of Birmingham, Birmingham, UK

**Keywords:** Huntington disease, disease progression, health costs, Markov process

## Abstract

We develop a Huntington’s disease (HD) progression model and integrate this with a novel economic model, accounting for the major factors of the HD’s societal cost. Data from the Enroll-HD observational study were used to fit a continuous-time hidden Markov disease progression model, which identified five distinct states. The number of disease states was determined using a cross-validated maximum likelihood approach. A novel data augmentation method was used to correct the biased life expectancy of the progression model. Multiple sources of cost data were then mapped to Enroll-HD variables using expert experience. A simulation of a synthetic patient population was used to show the feasibility of the approach in estimating population costs and the impact of hypothetical intervention scenarios. Our results confirm that early cognitive decline, which is not captured by the total functional capacity score currently used by clinicians but flagged up in HD integrated staging system, can be quantified from participants’ visits. Finally, the results of the UK cost modelling show that indirect costs of HD such as state benefits and lost gross domestic product contribution could be the driving factors for the societal cost, over and above health and social care costs.

## 1. Background

Huntington’s disease (HD) is a neurodegenerative genetic disorder resulting from an expanded cytosine-adenine-guanine (CAG) repeat in the huntingtin gene that is clinically characterized by involuntary movements, cognitive decline, and behavioural changes [1]. The combination of these symptoms eventually contributes to significant decline in functional capacity that affects both activities of daily living (e.g. eating, bathing) and instrumental activities of daily living (e.g. managing medication and finances) [2].

### 1.1. Decision problem

The aim of this work is to develop an HD progression model based on an objective data-driven assessment of disease states and integrate this with an economic model, accounting for the major factors of the societal cost of HD. A robust data-driven model of HD progression offers many potential uses, including population modelling, intervention evaluation, patient characterization and selection for clinical trials, diagnostics and outcome measurement. Our main objective is to have a progression model that can be linked to cost models, allowing societal cost analysis of disease progression. Potential applications linking to quality adjusted life years (QALY^1^) models and the impact of interventions can also be foreseen.

### 1.2. Motivation

Accurately modelling the progression of HD in relation to its economic impact is up to now still an unfulfilled need. Published models have concentrated exclusively on either disease progression modelling or costs modelling and may have required assumptions about the available patient data that might not be satisfied.

The progression of HD has historically been characterized in terms of the Shoulson–Fahn stages, which in turn are based on the total functional capacity (TFC) score [3]. There are several reasons why this is not a satisfactory model of HD progression, particularly when one wishes to model the potential economic impact of new therapies. Firstly, as the TFC score focuses on functional measures, it does not measure the gradual cognitive decline associated with HD, particularly early in the disease. Secondly, TFC is a function of a set of symptoms at a snapshot in time, regardless of the history of those symptoms. As such, it is possible for the TFC score, and therefore the Shoulson–Fahn stage, to improve at some point in time (e.g. owing to treatment or variation in functional capacity for reasons not related to HD), while it is known that the underlying biological progression of the disease does not improve, and there are no treatments or therapies known to slow or reverse progression of the disease.

### 1.3. Related work

A recently proposed alternative HD integrated staging system (HD-ISS) by Tabrisi *et al*. [4] used a series of large datasets with information on subgrouping in terms of age and CAG repeat size, and expert clinical consensus to devise a new staging system with four stages starting with 0 for individuals with the HD genetic mutation without any detectable pathological change, followed by stage 1 marked by measurable indicators of underlying pathophysiology, a detectable clinical phenotype in stage 2, and finally decline in function in stage 3. We discuss the differences in assumptions with our model in §4.

Wijeratne *et al*. [5] developed a temporal event-based model for inferring timelines of biomarker events in progressive diseases. They used a much smaller dataset than we did in terms of the number of individuals and included magnetic resonance imaging data. Imaging data were not available to us, and we see advantages in developing a model that does not use imaging data, which is expensive to create, slow to process, needs special permissions to obtain, needs more storage, etc. Their model also assumed that all the biomarkers were independent. We have not made such an assumption, as discussed in §2.2.

Most related to our model is previous work by Sun *et al*. [6], and Mohan *et al.* [7] that used a data-driven continuous-time hidden Markov model (CTHMM) for HD progression, with Bayesian latent variable analysis for deriving the features. Their model produces nine distinct states and is based on the data from several HD studies, including Enroll-HD dataset. We will discuss some of the assumptions of that model and how our model differs in §4.

In this work, we create a data-driven probabilistic HD progression model that allows us to calculate individual patient trajectories and estimate incurred societal cost for simulated interventions. Based on the UK-based publicly-available health and social care costs, we demonstrate how to estimate the societal costs associated with each disease state, and therefore how utilities, costs and future treatments can be incorporated to show the incremental outcomes among novel therapies.

We deliberately use the word ‘state’ for our progression model rather than ‘stage’ to avoid confusion with the Shoulson-Fahn/HD-ISS stages.

## 2. Methods

### 2.1. Disease progression model selection

Simple supervised models such as linear regression are not an option, as there is no directly observable state of the disease over time.

The progression of HD can be thought of being essentially a continuum of degeneration. To be able to model it and trace the results, we use the following simplifications.

As our aim is to be able to describe progression using measurable scores and to assign costs to it, we use state-transition modelling [8] which describes conditions that individuals are in as ‘states’, and likelihood of moves between the states as ‘transition intensities’ (the continuous-time counterpart of ‘transition probabilities’).

The spectrum of the disease is determined by carrying the huntingtin gene with 36 or more CAG repetition. We exclude family controls and genotype-negative individuals, so that we are only modelling the progression of HD. Our model has a lifetime horizon,^2^ and the data are corrected for life expectancy bias.

We chose a CTHMM for disease progression. This has been applied previously for modelling HD progression [6,7] and is consistent with International Society for Pharmacoeconomics and Outcomes Research-Society for Medical Decision Making (ISPOR-SMDM) modelling good research practices [9] and Brennan *et al*.’s taxonomy of model structures for economic evaluation of health technologies [10]. Markov models have also been applied for modelling other neurodegenerative diseases that progress in stages, such as Parkinson’s disease [11]. While ‘memoryful’ techniques are starting to be used to model disease progression [12], tractability and explainability of results still present a challenge. As our model is data-driven, we assume that there is an underlying biological disease process that is not directly observed, so hidden Markov models are appropriate. We are assuming that the underlying disease, characterized by the hidden states and a transition matrix, is always progressing, but the model has the flexibility such that observed clinical variables over time can still go up and down as the disease progresses. A continuous-time model also accommodates the fact that the patients’ visits are not equally spaced in time in the Enroll-HD dataset [13] that we used. It is worth noting that the number of states used is driven only by the input data, in the sense that inclusion of any further states was less likely. An overview of the CTHMM is given in the electronic supplementary material.

### 2.2. Data source

Data used in this work were generously provided by the participants in the longitudinal Enroll-HD study and made available by CHDI Foundation, Inc. Core datasets are collected annually from all research participants as part of this multi-centre longitudinal observational study. Data are monitored for quality and accuracy using a risk-based monitoring approach. All sites are required to obtain and maintain local ethical approval.

We used the Enroll-HD Periodic Dataset (PDS6) 2022-11 R2 containing data on *n* = 25 550 participants and a previous version from 2018 (PDS4) (*n* = 16 030). The overview including basic descriptive statistics of the population in PDS6 is given in [14]. All the details on data pre-processing and variable selection are given in the electronic supplementary material.

The progression model needs to consider functional, behaviour, motor and cognitive ability. After applying the pre-processing steps, 12 variables shown in table 1 were selected to be used as the inputs to the progression model. We then applied a grouped principal component analysis (PCA) to reduce these to a smaller set of less correlated features.

**Table 1.** List of selected Enroll-HD variables included in the progression model.

category	variable name	description	form
behavioural scores	exfscore_trans	(log-transformed) executive function score	problem behaviours assessment—short (PBA-s)
	irascore_trans	(log-transformed) irritability/aggression score	problem behaviours assessment—short (PBA-s)
	aptscore_trans	(log-transformed) apathy score	problem behaviours assessment—short (PBA-s)
functional scores	fascore_trans	(log-transformed) functional assessment score	UHDRS Functional Assessment Independence Scale (function)
	tfcscore	total functional capacity score	UHDRS total functional capacity (TFC)
	Indepscl	independence score	UHDRS Functional Assessment Independence Scale (function)
cognitive scores	swrt1	stroop word reading test total correct	cognitive assessments (cognitive)
	sit1	stroop interference test total correct	cognitive assessments (cognitive)
	sdmt1	symbol digit modality test total correct	cognitive assessments (cognitive)
	scnt1	stroop colour naming test total correct	cognitive assessments (cognitive)
	verfct5	verbal fluency test (category) total correct	cognitive assessments (cognitive)
motor score	motscore_trans	(Log-transformed) Motor assessment score	UHDRS motor diagnostic confidence (motor)

### 2.3. Correction of life expectancy bias

The preprocessed Enroll-HD dataset has very few recorded dates of death (about 5% of patients), so that the survival data are right-censored for the majority of patients. Such a high level of right-censoring leads to a bias, in the form of a large overestimation of life expectancy.

We could have only included patients whose dates of death are known, but this would have reduced our dataset size drastically and would have led to underestimation of the life expectancy, owing to the selection bias of keeping this subset of patients. Another option would have been to only try to model progression up to motor onset, as we would have more data for this event, but that would not meet our goal of characterizing the societal costs throughout a patient’s lifetime. Instead, we introduced an artificial upper bound for the death age of patients with no recorded death to address this bias. We picked an upper bound that resulted in a mean age at death for HD patients of 63.9 years based on data from the Norwegian Cause of Death Registry [15]. Details of this method can be found in the electronic supplementary material.

### 2.4. Number of states selection

To select the optimal number of states for the CTHMM, we used a similar method to that of Sun *et al.* [6] and Mohan *et al.* [7], but we improved robustness via cross-validation during training. The details are provided in the electronic supplementary material.

### 2.5. Progression model fitting

We used the R package MSM^3^ [16] to fit the CTHMM.

The observations at visits are treated as snapshots in time, while recorded dates of death, along with artificial birth observations, are treated as direct observations of the start time of states $n_{s}$ and 1, respectively. We disabled the estimation of the initial probabilities, since as discussed above we can assume the initial state is 1 for all patients. The artificial death upper bounds for correction of the life expectancy bias are treated as interval-censored observations of a transition from state $n_{s}-1$ to $n_{s}$ at some time between the last visit and the upper bound.

We require the observed features used in the CTHMM to have low correlation,^4^ as the progression model fitting method assumes that the observed features are independent. Since we are using a grouped PCA based on age brackets, the principal components (PCs) are guaranteed to be uncorrelated within each age bracket but not guaranteed to be uncorrelated in each fitted disease state. We used an iterative algorithm for fitting the progression model as follows:

- split the visit data into age brackets and perform PCA on each age bracket separately;
- keep the first $n_{\text{PCs}}$ PCs and use these as the observations for fitting the CTHMM model; and
- check the correlation between the PCs for each fitted disease state, where the fitted disease state is determined from the fitted CTHMM using the Viterbi algorithm. If the absolute values of the correlations are all below some threshold (we used 0.2^5^), stop. Otherwise, recalculate the PCs using the fitted disease state as the grouping. Return to step (ii).

### 2.6. Estimation of costs

**Figure 1.**
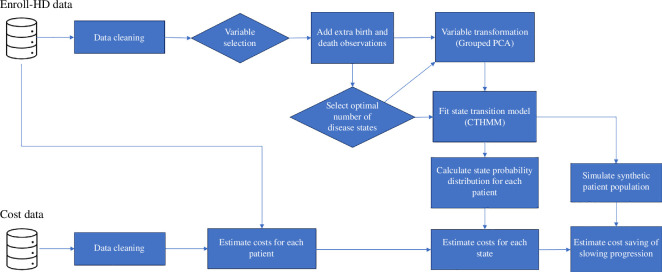
An overview of our model consisting of disease progression (continuous-time hidden Markov model) and estimated cost per state.

Once we established the optimal number of disease states and the model for progression, we then estimated costs (see figure 1). In this initial feasibility work, for simplicity, it is assumed that costs are constant while a patient is in a particular state (we ignore inflation and potential cost escalation).^6^

There are many societal costs incurred owing to a patient’s HD. Some of these have related data in the Enroll-HD dataset—specifically, there is data on which pharmacological and non-pharmacological therapies each patient was receiving over which period, and at what dosage/frequency, and we know whether the patients live at home or in a care home at the time of a visit.

Clearly the costs incurred by each individual, even if in the same disease state, will vary. The cost models derived here must support population simulations of overall cost. Hence, where appropriate, suitable probabilistic models for individual costs, with realistic variability as TFC and other measures change, are derived to support the associated Monte-Carlo simulations described in the next section.

#### 2.6.1. Pharmacological and non-pharmacological therapy costs

We used data from the NHS prescription cost analysis for 2022/2023 [17] to match medications in Enroll-HD to a cost by scaling the unit costs according to the patient’s dosage.

For non-pharmacological therapies, we used the social care unit costs 2022 [18] data to estimate the cost of therapy sessions.

#### 2.6.2. State benefit costs

We used data from the UK Government website (https://www.gov.uk) on the benefits available in England and Wales, specifically universal credit (UC), personal independence payment (PIP) and employment support allowance (ESA). We obtained the data in January 2024, so our analysis represents the financial support available at that time. As government policy changes, HD patients may start or stop being eligible for various benefits, and the associated amounts will be subject to change. The rest of this article reflects the state of UK benefits at the time of obtaining the data. Details on how different benefits are estimated are given in the electronic supplementary material.

#### 2.6.3. Lost contribution to gross domestic product

The approach to estimating a patient’s lost contribution to gross domestic product (GDP) is detailed in the electronic supplementary material. This is a function of salary, which is estimated adjusting for the ages of Enroll-HD patients. We assume that when occupation status drops from 3 (normal) to 2 (reduced capacity for usual job), the patient’s salary reduces by half, and when occupation status drops to 1, the patient’s salary is 0. We randomly generate nominal salaries for the Enroll-HD patients at study baseline by assigning a percentile value (i.e. a value between 0 and 1) to each patient randomly from a uniform distribution, then using the salary distributions in [19] to convert this to a random salary. Although we do not expect an individual’s salary to be accurate, this should give a representative distribution of salaries when done across all patients in the Enroll-HD training set. Note that we are randomly simulating what the patients’ salaries would have been in the absence of HD, and then decreasing that as the disease progresses as described above.

We assign no loss of contribution to GDP for patients who are at or above the state pension age, as they would not be contributing to GDP anyway.

#### 2.6.4. Cost of care home/care worker/partner stopping work

**Figure 2.**
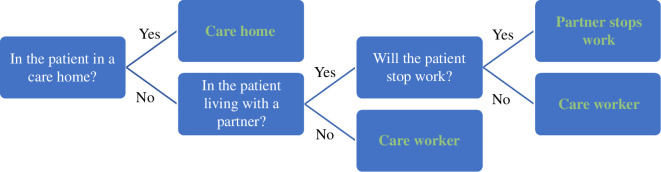
Decision tree for whether a patient uses a care home, care worker or their partner stops work when their disease severity is such that they need this level of care.

There comes a point in HD progression at which the patient cannot look after themselves at home, and so either their partner needs to stop work, or they need the support of a care worker. The earliest TFC score agreed in discussions with specialist Huntington disease advisors for when this happens is 8, and by a TFC of 6 all patients are expected to be needing such care or be in a care home. The logic for determining who provides a patient’s care is shown in figure 2. This logic is evaluated separately for each visit. We determine whether the patient is living with a partner from the ‘maristat’ variable in Enroll-HD. We do not have data on whether the patient’s partner will stop work, so we assume that this is random with a probability of one-half. Note that this represents whether the partner will stop work or bring in a care worker when the HD patient needs someone to care for them during the day. As explained above, the patient’s TFC at the time this decision needs to be made will be at most 8.

The expected hourly rate of a care worker is calculated using data from the Health and Social Care Unit Costs [18]. There is local authority funding available for the cost of a care worker, but PIP is also intended to support this cost, so the local authority funding available for care workers is reduced by the amount of PIP claimed. Further details are in the electronic supplementary material.

#### 2.6.5. Simulation of hypothetical treatment scenarios with a synthetic patient population

By way of illustration, to simulate hypothetical treatment scenarios, we used a multi-step process. First, we simulated baseline costs as follows:

- simulate disease progression for a synthetic patient population using the fitted CTHMM;
- sample total costs (per month) using the fitted total cost distribution (generated from the actual patient Enroll-HD data as a function of their fitted state, using the individual cost models derived in this paper conditional on the measured data for that patient); and
- sample individual costs consistent with the total cost using the Dirichlet method (see the electronic supplementary material for details of this method).

We considered a hypothetical treatment scenario in which the transition between two disease states is delayed by some time Δt (details in the electronic supplementary material).

In sampling the total costs for each disease state of each synthetic patient, we modelled the cost as constant for the duration of the disease state, and we sampled only a single U[0, 1] random number for each synthetic patient, and used this to generate random costs at the same quantile level for each disease state in their progression.

#### 2.6.6. Model validation

We have run the probabilistic modelling procedure as described above originally with Enroll-HD PDS4 (2018) and costs from 2020/2021 and then independently reran it with the PDS6 (2022) and costs from 2023/2024. In the space of 4 years, the number of participants grew from 16 000 to 25 000 and the number of visits (and data points used) almost doubled. The fact that the number of states, sojourn times, PCA, state distributions and cost distributions did not significantly change shows that the model is robust with regard to size and change in data.

## 3. Results

In this section, we describe the results, firstly on determining number of states and fitting the disease progression model to the Enroll-HD data, and then on the estimated economic costs of the UK HD population across different states of disease.

### 3.1. Feature selection

**Figure 3.**
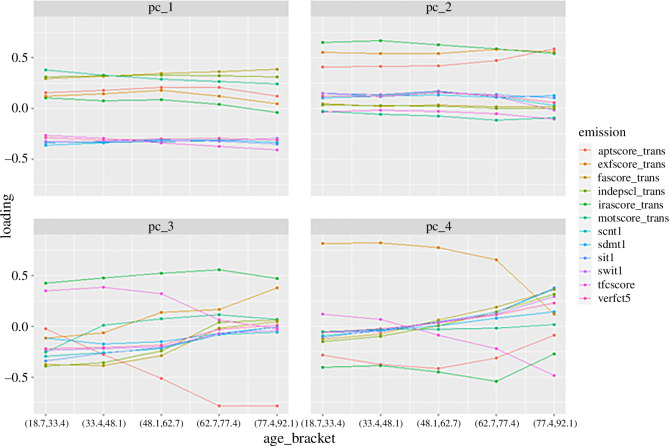
Principal component loadings for each age bracket.

The loadings for the first four PCs in the grouped PCA (as described in the ‘Data preparation’ section from the electronic supplementary material) are shown in figure 3.

The loadings for the first principal component (PC1) are fairly consistent across age brackets, with only a gentle drift. The loadings for the second principal component (PC2) have a more noticeable drift across age brackets in the loadings for apathy score, executive function score and irritability/aggression score. Note that PC2 is driven by these three ‘problem behaviours’ variables, while PC1 is driven by the other variables, which are all strongly correlated with each other. The third principal component (PC3) and fourth principal component (PC4; and higher PCs) show so much variation in the loadings across age brackets that they cannot be included in the progression model, as it is not clear that the scores would be meaningfully comparable between visits. As our method found that PCA on the whole dataset could not be justified, we only did an *a priori* examination of the percentage variance explained with the patients grouped by age bracket. The first PC explains 59–69%, and the second PC a further 10–15%, which are both similar to the findings in [20].

Based on the above observations, we selected the first two PCs to use in the progression model.

### 3.2. Number of states selection

**Figure 4.**
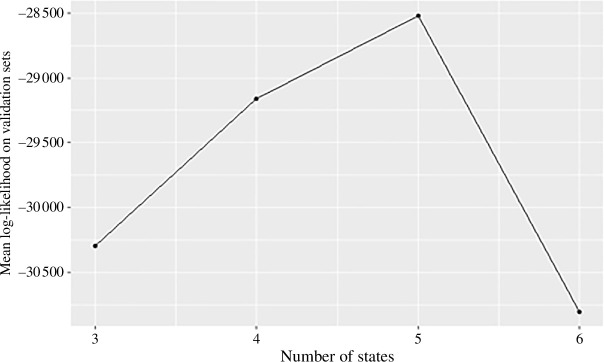
Results of fivefold cross-validation method for selection of the number of states, in which five states gives the maximum mean likelihood over the five validation sets.

The results of the fivefold cross-validation are shown in figure 4, showing that five states (four hidden states and death) give the highest mean likelihood.

### 3.3. Progression model fitting

**Figure 5.**
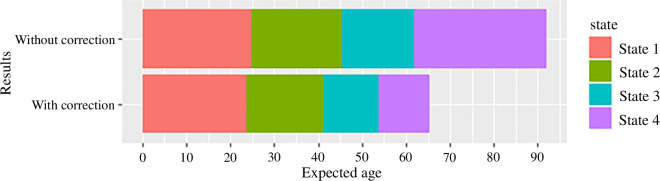
Fitted mean sojourn times in each living disease state, with and without correction for life expectancy bias.

The fitted mean sojourn times of the progression model with and without the correction for life expectancy bias are shown in figure 5. The duration of the final state, in particular, is significantly reduced with the correction and appears much more consistent with our expectation of the life expectancy of HD patients.

**Figure 6.**
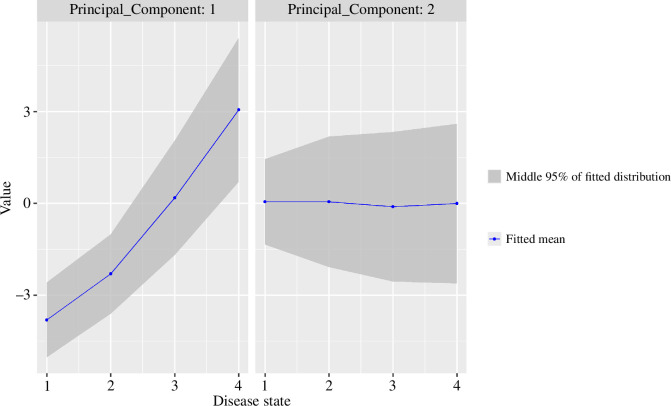
Fitted emission distributions.

The fitted emission distributions (i.e. the fitted distributions of the observations used in the progression model, which are the PCs) are shown in figure 6. We can see the general trend of PC1 as the disease progresses. PC2 has very little change in the mean but has increasing variance as the disease progresses. PC2 is dominated by the behavioural scores (irritability/aggression, executive function and apathy), but these variables also make a small part of PC1. The flat mean for PC2 shows that the increase in mean of these variables is captured by PC1, while the increase in variance of these variables is an independent effect that is captured by PC2. For example, a patient with a constant moderate irritability may be at an earlier disease state than another patient with the same average irritability but who shows a lot of variation over time between not at all irritable and highly irritable. Another possible interpretation of this is that while the decline in the cognitive, motor and functional domains is fairly consistent across all patients, some patients will show no decline in these behavioural variables while others will show a very significant decline.

**Figure 7.**
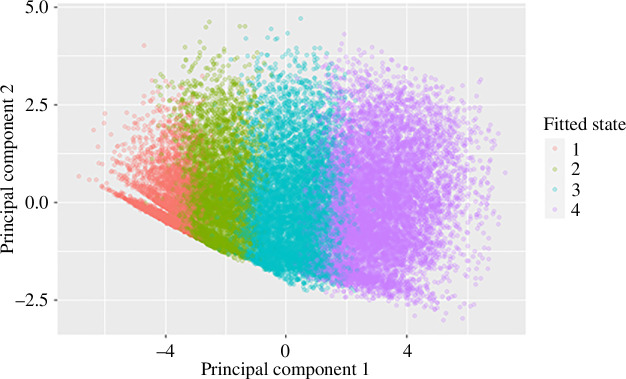
Principal component scores and fitted state of Enroll-HD patient visits.

We also examined the most likely state sequence for each patient in the Enroll-HD training set and examined their PC scores to check for patterns or correlations. The PC scores are shown in figure 7. The Viterbi algorithm was used to calculate the most likely state sequences from the fitted progression model. The main progression of the disease can be seen moving from left to right in the plot. Some lines are appearing, particularly in states 1 and 2. This is most likely because there is saturation of some of the assessment scores at a healthy level; e.g. TFC maxes out at 13, independence score maxes out at 100.

The Pearson correlation coefficients between the scores of the first two PCs were all found to be below our threshold of 0.2, hence there was no need to repeat iterations in the algorithm outlined in the Methods section under *Progression model fitting*.

**Figure 8.**
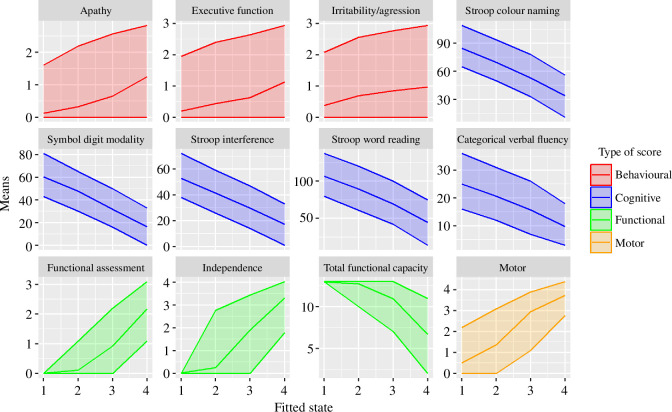
Distributions of raw observations grouped by fitted disease state. The solid line shows the mean, and the grey band shows the middle 95% of the distribution (i.e. variation across the population, not a confidence interval).

We also examined the distributions of the raw observations grouped by fitted disease state—this is shown in figure 8. We can see that several variables, particularly the cognitive assessments, have a much clearer decline between states 1 and 2 than the TFC and motor score. There are three key observations we can draw from this: firstly, TFC does not capture the cognitive decline (worsening of the cognitive observation scores) that affects patients in early states of HD. Secondly, it appears that motor score rate changes with the transition from state 1 to 2 and then again from state 2 to 3. The biggest rise in functional assessment roughly coincides with the transition from state 1 to 2. Thirdly, different behavioural scores change rates change significantly between the states, irritability/aggression between 1 and 2, apathy between 2 and 3 and executive function (overall problems in behaviour) across state 3.

### 3.4. Comparison of fitted state with Huntington’s disease-integrated staging system stages

**Figure 9.**
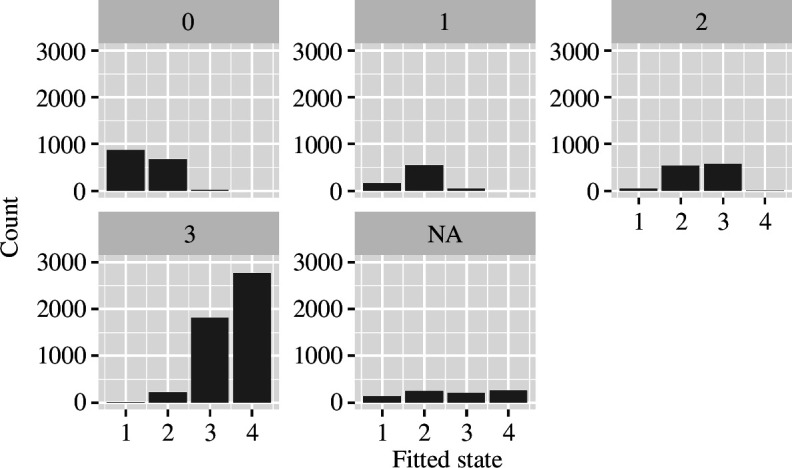
The patients' baseline visit HD-ISS stage (from Enroll-HD) is given in the title of each subplot, and our model’s fitted states for the same patient are given on the *x*-axis.

We compared our model’s fitted states at their first visit to the patients’ HD-ISS stages [4] imputed values available in the newer dataset (PDS6). In figure 9, one can see that a stage includes mostly patients in two neighbouring states (stage 0 covers patients in states 1–2, stage 2 patients in states 2–3, and stage 3 patients in states 3–4), with the exception of stage 1, for which the majority of patients are in state 2.

### 3.5. Estimation of costs

**Figure 10.**
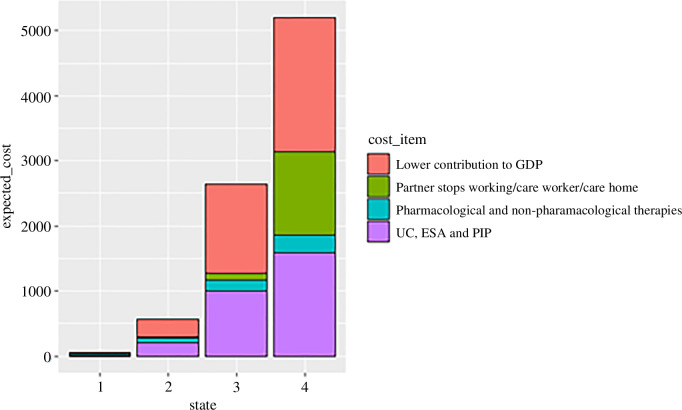
Expected monthly costs in GBP in each category by disease state.

The estimated mean monthly costs in each category we considered are shown in figure 10 and tabulated in table 2.

**Table 2.** Estimated monthly costs (GBP) for each disease state in various categories.

disease state	UC+ESA + PIP	pharmaco/nonpharmaco therapies	lower contribution to GDP	partner stops working/care worker/care home
1	12	22	14	3
2	203	79	268	4
3	1006	158	1378	106
4	1586	271	2056	1285

**Figure 11.**
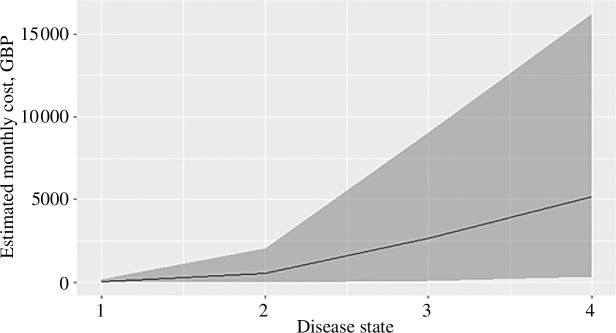
Estimated individual monthly total cost distribution for each disease state across the population. The solid line shows the estimated mean, and the grey band shows the middle 95% of the fitted distribution.

We estimated individual monthly total costs of all patients at each given disease state. Figure 11 shows the means and 95% confidence intervals of the resulting marginal distributions of the individual costs across the population for each disease state. As expected, costs vary significantly between individuals, but the total population cost burden is driven by the population mean.

### 3.6. Simulation of hypothetical treatment scenarios

Using a hypothetical treatment that delays entry from state 2 to state 3 (which approximately corresponds to motor onset) by 10 years, we found with our simulation that the mean cost saving per patient was of the order of £100 000 on average over the patient’s lifetime (including ‘costs’ incurred after a patient’s death if it was before retirement age owing to lost contribution to GDP). The simulation included 10 000 synthetic patients.

While such results demonstrate the feasibility of using our approach as the basis for cost analysis, it should be recognized that a full picture should also include, for example, lost opportunity costs for family members, and such contributions may be expected to be significant. However, it is insightful to examine the key factors that result in the saving. The simulated delay means that in fact more costs are incurred in state 2 than the baseline, while costs in later stages move to the right. However, the key saving comes from a reduction in lost contribution to GDP, which ends at retirement age and therefore is incurred for less years than baseline. While this results in a net saving, it does illustrate the complex and often non-intuitive factors that give rise to changes in costs.

### 3.7. Summary

By fitting a CTHMM to Enroll-HD data, we determined that the number of disease states most consistent with the data was five. The average durations of the first four states were estimated as (23.6, 17.6, 12.7, 11.4) years (the final state is death). Examining the fitted progression model showed a clear distinction between patients who had a high TFC score and were still highly functional and patients who also had a high TFC but showed clear signs of early cognitive decline, before motor onset (which is broadly consistent with the findings in [4] and [6]). We also found that behavioural scores change rate accelerated with transition from one state to another, and that motor decline was offset with the transition from state 1 to 2.

Regarding societal costs, among the highest average costs associated with the most advanced disease state while living were loss of the patient’s contribution to GDP (£2056 per month), state benefits claimed by the patient (£1557 per month) and the cost of either the patient’s partner stopping work or the cost of a care worker or care home (£1285 per month). While there are additional factors that could be included (e.g. opportunity costs for children and young people growing up in HD families), and the analysis is currently limited to the UK, this approach does show the principles of how a comprehensive costing model could be developed for different scenarios.

## 4. Discussion

### 4.1. Overview of key conclusions

Our data-driven modelling approach has been used with the Enroll-HD data to infer the number of disease states (five) and fit a state-transition progression model. The progression model has been linked with a cost model to allow predictions and potential ‘what-if’ cost-benefit analyses to be performed. Furthermore, the approach has corrected a life expectancy bias introduced owing to limitations of the dataset used and predicts state sojourn times consistent with understanding of HD. These outcomes appear to support the use of such a model for HD progression, noting that care must be taken to ensure the assumptions of this model are respected.

Analysis of the emissions conditional on the fitted state shows that the measurements appear to fall into two groups, with only one group appearing to capture a decline between states 1 and 2. Examination of these groups suggests that cognitive decline and change in behaviour are evident when moving from state 1 to state 2, while functional symptom onset is more evident when moving from state 2 to 3. It is noted that this insight has been generated from a purely data-driven approach and hence is considered objective. Such insights must be critically re-examined if the progression model is refitted using additional data like in [4,6,7], but we have validated results on two non-consecutive snapshots of Enroll-HD periodic datasets (PDS4 and 6). Despite the differences in derived numbers and definitions of states or stages, this data-driven capture of early cognitive decline appears consistent with the findings presented in [4,6,7].

The stages in [4] are inferred using data available based on two pre-decided measurements per stage. This strategy is tested through analysis of age-related probabilities of landmark and stage progression, and this does support the selected measurements for pathological and functional decline. However, apart from one subpopulation (younger individuals with high school education), we interpret the clinical decline to be driven by only one of the selected measures (Total Motor Score (TMS)) with the other (Symbol Digit Modality Test (SDMT)) being far less likely to trigger the landmark than TMS, so the significance of the inclusion of SDMT on the results is unclear. We also note that ‘detectability’ is a function of test sensitivity, so it is not obvious that clinical detectability would in all cases predate functional detectability^7^.

In comparison to the progression models from [6,7], our model has fewer states: five compared to nine, and clearly this is a key difference that requires some analysis. We note that our datasets do differ, with PDS4 being a subset of that in [6], which could have an impact. However, there are additional differences in our approach which could also be relevant:

- we use a similar method for selecting the number of states but attempted to introduce increased robustness by using cross-validation;
- we did not allow instantaneous ‘skipping’ of disease states;
- we included a state corresponding to death, allowing us to improve the state sojourn time estimates using other data on the life expectancy of HD patients. Note that the dataset used in [6,7] could also lead to a life expectancy bias, and it is not described how this was handled. The broader observational datasets used appear to cover more longitudinal measurements typically, which would be expected to have a beneficial effect in this regard, although terminal phase HD data is noted as being sparse; and
- for feature selection, Sun *et al*. [6] used Bayesian latent variable analysis and used the leading three latent factors from each of the motor, functional, and cognitive domains. Our approach did not apply this constraint on domains and used only two PCs. This, coupled with only five states in our progression model, means we have significantly fewer parameters than Sun *et al*.’s model, but also allowed confirmation that our model assumptions were respected.

The link to cost models gives the potential for better insight into the differing sources of cost and their comparison, as well as the key drivers. We have also demonstrated the application to ‘what if’ cost–benefit analyses of potential interventions, based on hypothetical impact on the disease progression. This we believe demonstrates a solid basis for cost modelling that combines data and subject matter expertise and can be extended to other countries and with additional sources of cost.

In addition to population analysis, our approach also allows for estimation of a patient state from their measured data. This opens up the potential application of our progression model in other contexts, such as patient characterization and trial selection, and outcome determination from measured data (e.g. additional time in a particular state). Furthermore (in combination with a suitable model extension), it could be applied to other metrics such as QALY prediction over patient lifetime.

### 4.2. Limitations of Huntington’s diesease progression model

The method for choosing the number of disease states to include in the progression model is based on evidence in the Enroll-HD datasets used. The result that five states gave the highest likelihood was validated using the periodic sets in two different data releases (2018 and 2022). However, we cannot confirm that the result is repeatable with other datasets. Refitting the progression model in the future as more data becomes available (e.g. including imaging or wet biomarkers or clinical trials data) will allow this to be investigated.

Since we excluded visits from the training data that had any of the required observations missing, the usable data for a patient was 2.88 visits on average (with standard deviation of 1.92) spanning 2.35 years on average (with standard deviation of 2.32). Rerunning the model in the future with more longitudinal data per patient will demonstrate the importance of this effect, although we believe that the large number of patients mitigates this issue.

### 4.3. Limitations of cost model

Our mapping from Enroll-HD variables to costs was done based on the expert opinion of a few advisers and used to show the feasibility of such an approach. Ideally, this would be done based on a large dataset—the relevant benefits and funding, etc. that a patient is receiving would be recorded as part of a visit. While we have tried to capture the uncertainty owing to variation between patients, we were not able to quantify the uncertainty in the underlying assumptions of the cost model. Some of these assumptions are very simple, such as how we assume earnings decrease with disease progression and how likely patients’ partners are to stop work. Future work should concentrate on improving these aspects.

### 4.4. Future work

The results of this work, together with those in [6,7], suggest that there is real potential in using data-driven progression approaches to provide detailed insights into HD disease progression. Hence, a natural next step is further work to investigate and compare the different modelling approaches and datasets used, and expand the data used to fit the progression models.

Including other longitudinal data (e.g. imaging and wet biomarkers, and especially any available clinical trials data) and rerunning the model would also allow further validation of our model.

Updating, contrasting and combining the existing models in the light of new data will take some time, and we think that this work is a part of that iterative process to achieve the vision—a stable staging system grounded in data, that is flexible and robust enough to be used with confidence in clinical practice, in clinical trials and in designing and estimating benefits of new therapies and interventions.

Now that a feasible cost modelling methodology has been established, the associated cost models could be developed further to include additional elements, for example lost opportunity costs in the patient’s family, and to replace the simple assumptions around salary and partners stopping work with more robust approaches. The work can also be extended to link to models for other metrics, for example QALY.

## Data Availability

Publicly available datasets (Enroll-HD-Periodic-Dataset-2018-10-R3 and Enroll-HD-Periodic-Dataset-2022-11-R2) in this study can be found at https://www.enroll-hd.org/for-researchers/access-data/. Relevant code for this research work is stored in GitHub [21] and has been archived within the Zenodo repository at [22]. Supplementary material is available online [23].

## Footnotes

^1^
QALY—quality adjusted life years is a generic disease burden measure, an index of survival that is adjusted to account for the patient’s quality of life during the time of intervention. QALYs incorporate changes in both quantity (longevity/mortality) and quality (morbidity, psychological, functional, social, and other factors) of life and is used in economic evaluation to assess the value of medical interventions.

^2^
With the exception that the age for individuals under 18 years in Enroll-HD is represented by the value ‘<18’

^3^
This package maximizes the progression model likelihood using standard optimization algorithms, as implemented in the ’optim‘ function in R (minimizing the minus log-likelihood). We used the default ’BFGS‘, a quasi-Newton optimization method. We used the principal components (PC) as the observations for the progression model and modelled these as independent normal (Gaussian) random variables for each disease state.

^4^
This was an assumption of the model in the MSM package that we used, which has influenced our choices of variables and transformations to use in the model. Ideally, we would use more variables and account for correlations directly in the model likelihood rather than restrict ourselves to Gaussian approximations and PCA, but that would require development of tools for fitting CTHMMs with correlated observations, which was out of scope for our project.

^5^
This value represents a trade-off between uncorrelated variables and the complexity of the method—the lower we set this value, the more iterations of re-grouping are required and the more complex the method becomes. Some experimentation with more iterations of re-grouping showed that using lower thresholds made little difference to the results.

^6^
Net present value (a way to compare cash flow for different courses of action, accounting for inflation together with associated ’what-if’ analysis of cost escalations) can be readily included in an extended cost model and would be required for any estimation of future absolute costs, however here we focus on the demonstration of the model feasibility and hence did not include this aspect.

^7^
Using the currently available data, including clinical examination and rating scales, some individuals will clearly decline before they reach the threshold of diagnosis, and some individuals will decline much slower, after the threshold is reached. This reduces the validity of ’clinical detectability/diagnosis’ altogether, except in the situation of differential diagnosis (to establish if the presented illness is HD or something else, which is a rare situation in HD because of the genetic marker).
